# Nasce um corpo: transformações contemporâneas no conhecimento produzido entre a Sociologia e a Saúde

**DOI:** 10.1590/0102-311XPT096623

**Published:** 2024-07-29

**Authors:** Marília Moschkovich

**Affiliations:** 1 Faculdade de Filosofia, Letras e Ciências Humanas, Universidade de São Paulo, São Paulo, Brasil.

**Keywords:** Teoria Social, Sociologia, Gênero, Relações Raciais, História da Medicina, Social Theory, Sociology, Gender, Race Relations, History of Medicine, Teoría Social, Sociología, Género, Relaciones Raciales, Historia de la Medicina

## Abstract

De que modo os fenômenos sociais entendidos como pertencentes à esfera da saúde provocaram a Sociologia nas últimas décadas? Este estudo parte dessa pergunta para articular Sociologia e Saúde, propondo uma reflexão sobre a maneira como o conhecimento pode avançar na interseção entre essas duas áreas. O texto é organizado em três seções: na primeira, oferece uma breve reflexão sobre a noção de “problema sociológico contemporâneo”, indicando as questões da Sociologia da Saúde que podem ser tomadas como vetores para essa indagação; na segunda, examina algumas contribuições da Sociologia da Saúde, sobretudo a partir da segunda metade do século XX, pontuando a maneira como essa área precisou organizar soluções e reconfigurar problemas sociológicos para dar conta de fenômenos contemporâneos; e por fim, na terceira seção, o texto propõe novas perguntas para pensar a saúde como problema sociológico contemporâneo no contexto político atual.

## Abstract (en)

In what ways have the social phenomena understood as belonging to the sphere of “health” provoked Sociology in recent decades? This essay takes this question as a starting point for linking Sociology and Health, proposing a reflection on how knowledge can advance at the intersection between these two fields of knowledge. The article is structured in three sections: in the first, a brief reflection on the notion of “contemporary sociological problems” will be presented, indicating the questions of the Sociology of Health that can be taken as a vector for this inquiry; in the second, it examines some contributions of the Sociology of Health, especially from the late 20th century, indicating how this area has had to organize solutions and reconfigure sociological problems to deal with contemporary phenomena; and in the last section, it proposes new questions for thinking about health as a sociological problem in the contemporary political context.

## Resumen

¿De qué manera los fenómenos sociales entendidos como pertenecientes al campo de la “salud” han provocado la Sociología en las últimas décadas? Este ensayo parte de este interrogante para articular Sociología y Salud en una reflexión sobre cómo el conocimiento puede avanzar en la intersección entre estas dos áreas del conocimiento. Este texto se organiza en tres apartados. En el primer apartado se reflexiona brevemente sobre la noción de “problema sociológico contemporáneo” indicando las cuestiones de la Sociología de la Salud que pueden considerarse como motor para esta cuestión; en el segundo, se examinan algunas aportaciones de la Sociología de la Salud, especialmente de la segunda mitad del siglo XX, en las cuales señala cómo el área necesitaba organizar soluciones y reconfigurar los problemas sociológicos para abarcar los fenómenos contemporáneos; y, en el tercer apartado, se plantean nuevos interrogantes para pensar la salud como un problema sociológico contemporáneo en el contexto político actual.

A pergunta se repetiu durante as 12 horas em que estive no centro obstétrico. Minto, antes de entrarmos no centro obstétrico do hospital municipal do Campo Limpo (São Paulo, Brasil), eu já havia escutado a mesma algumas vezes: “*você é o quê dela?*”. Os olhares confusos de enfermeiras, enfermeiros, médicas, residentes e outros funcionários da administração do hospital denunciavam o desconforto diante da resposta: “*amiga, ué*”, eu respondia. Em certo momento, um dos enfermeiros ainda seguiu a conversa, insinuando de volta: “*Deve ser uma amiga muito próxima para ficar 12 horas aqui acompanhando indução de parto*”, ao que respondi, sem pensar duas vezes, que, “*ora, se minha melhor amiga estivesse parindo e eu não me dispusesse a estar junto quando ela precisasse, que espécie de melhor amiga seria eu?*”. Éramos, na verdade, uma equipe: a parturiente e seus dois companheiros e pais da criança, eu, meu namorado, o meu marido [pai da minha criança], e nossa outra melhor amiga, com seu ex-marido e atual casinho eventual [pai da criança dela]. Uma vila, talvez. A norma do hospital só permitia um acompanhante por vez para cada parturiente - o mínimo exigido pela lei. A troca de acompanhantes só poderia ser realizada em certo horário, a cada 12 horas.

O parto da pequena Nelly durou dois dias e um tantinho, quase batendo o meu recorde pessoal de ter parido em três dias sem indução nenhuma, alguns anos antes. Esse parto, que começou com uma melhor amiga como acompanhante porque nenhum dos dois pais do bebê falava português fluente e o medo inicial de sofrer violência obstétrica era grande, desafiava a norma para a qual o hospital e as equipes de saúde tinham se preparado e o que tinham executado a vida toda: a bebê tem dois pais, nenhum deles é brasileiro, e a acompanhante inicial, para cuidar do começo da indução e conversar com as equipes, é a melhor amiga, sendo intransigente quando necessário. De forma quase surpreendente, a burocracia hospitalar ruiu um pouco naqueles dois dias: na hora agá, depois de mais de 48 horas convivendo com aquela gestante, as equipes do centro obstétrico toparam chamar o segundo pai que esperava ansioso sem saber se veria sua filha nascer. Nelly nasceu numa tarde de outubro, amparada por toda a sua rede de cuidados, que extrapolava o sentido de “família”, e desafiava o regimento hospitalar.

Como esse, os demais fenômenos concretos da vida social rotineiramente desafiam as normas e categorias de entendimento do mundo já estabelecidas, e o que entendemos socialmente como pertencentes ao campo da saúde não são exceção. As próprias disputas em torno de como classificá-los, assim como em torno da categoria “saúde”, são evidências desse tipo de processo. Neste ensaio, distante da possibilidade ou intenção de esgotar o debate em torno de tais disputas e ressaltando ser um pequeno recorte relativo a um amplo e complexo campo de estudos, procuro observar de que maneira o interesse sociológico por essa gama de fenômenos provocou a Sociologia e permitiu compreensões complexas sobre pontos de tensão entre a experiência concreta e as normas sociais/visões de mundo anteriores. Essas questões orientam o este texto, organizado em três seções: na primeira, oferece-se uma breve reflexão sobre a noção de “problema sociológico contemporâneo”, indicando as questões da Sociologia da Saúde que podem ser tomadas como vetor para essa indagação; na segunda, examina-se algumas contribuições da Sociologia da Saúde, sobretudo a partir da segunda metade do século XX, pontuando a maneira como essa área precisou organizar soluções e reconfigurar problemas sociológicos para dar conta de fenômenos contemporâneos; por fim, na terceira seção, o texto propõe novas perguntas para pensar a saúde como problema sociológico contemporâneo no contexto político atual.

## Problema sociológico e problema sociológico contemporâneo

As relações sociais ordinariamente tensionam as categorias que as produzem e o conjunto de relações das quais são herdeiras. É nesse movimento que a Sociologia, enquanto espaço produtor de conhecimento sobre a vida social, se alimenta de novos problemas que, afinal, são dispositivos que produzem crises. As ferramentas desenvolvidas para atravessar certa experiência deixam de ser adequadas, produzindo um espaço de criação para o novo - como num parto, em que o sistema já ajustado de funcionamento do corpo grávido chega ao seu limite, e a única possibilidade de seguir existindo é nascer. Todo problema é, assim, um motor. Os sociológicos, em particular, são motores desse sistema de conhecimento do mundo que convencionamos chamar de Sociologia. Toda sociologia é do conhecimento, afinal, embora não exatamente como propôs Collins ^1^ em *Four Sociological Traditions* [Quatro Tradições Sociológicas], uma vez que não há sentido teleológico e nem necessariamente progressivo no desenvolvimento das Ciências Sociais e Humanidades ou da Sociologia como uma de suas disciplinas (e muito menos um sentido progressivo que toma a epistemologia europeia baseada na invenção do Ocidente como máximo do progresso, como lamentavelmente faz esse autor). Os problemas sociológicos são projetos de deslocamento, e esses contemporâneos lidam, portanto, com o que há de aparentemente inexplicável ou desafiador em cada época. Mas do que falamos quando nos referimos ao nosso contemporâneo?

Embora algumas formas de produzir Sociologia, assim como estruturas sociais que as atravessam, permaneçam semelhantes desde a segunda metade do século XIX − período que conhecemos como quando a Sociologia se funda enquanto disciplina, segundo autores como o próprio Collins ^1^, Giddens ^2^ e outros - é possível afirmar (com razoável certeza) que é no pós-guerra, a partir da década de 1950, que ela adquire a maior parte de seus contornos fundamentais do presente. O pesquisador holandês Johan Heilbron, em *The Rise of Social Theory* [A Ascensão da Teoria Social] ^3^, chama as Ciências Sociais e Humanidades como as conhecemos hoje de *disciplinary Social Sciences and Humanities* (Ciências Sociais e Humanidades disciplinares). Ele observa a maneira como a configuração disciplinar atual das Ciências Sociais e Humanidades se inicia na virada do século XIX para o XX, mas se consolida apenas após a Segunda Guerra. Um dos processos fundamentais apontados pelo autor para isso é a reconfiguração das universidades e a institucionalização das Ciências Sociais e Humanidades nos países centrais do capitalismo. No período, os Estados Unidos passaram a ser a principal potência econômica do mundo, o que implicou uma transformação das políticas de *soft power*, tanto aquelas promovidas por esse país quanto as promovidas por outros países que perderam o espaço de potências, como a França ou a Alemanha. Essas políticas se concretizavam principalmente por meio do incentivo à circulação internacional de pesquisadores e cientistas e financiamento dos departamentos de Ciências Sociais e Humanidades em universidades por todo o mundo.

Nesse contexto, completa Heilbron ^3^, é que as Ciências Sociais e Humanidades passaram a ser reconhecidas como espaço de produção de conhecimento voltado para a gestão estatal e a produção de políticas públicas. As áreas de pesquisa que se configuram hodiernamente, como as disciplinas de Ciência Política e Relações Internacionais, são casos exemplares disso, assim como a transformação da *political economy* em *economics*, acompanhada pela matematização de tal espaço disciplinar. É nesse sentido que opto, aqui, por trabalhar tendo esse marco - o pós-guerra, notadamente a década de 1950 - como início do que escolho chamar de “contemporaneidade” para a Sociologia e as Ciências Sociais e Humanidades em geral.

Utilizar esse marco para tratar da contemporaneidade neste texto não significa, contudo, limitar a Sociologia aos autores que produziram a partir dessa época. Da mesma maneira que um corpo que nasce é herdeiro das células que tinha enquanto feto, as ferramentas analíticas da Sociologia contemporânea são herdeiras de suas reinterpretações e usos (táticos, teóricos, políticos, científicos) dos ditos “clássicos” e de autores que contribuíram em períodos anteriores com o acúmulo coletivo da área - como fez Fanon ^4^ ao reinterpretar Karl Marx, Judith Butler ^5^ ao reinterpretar Simone de Beauvoir, ou ainda Lélia Gonzalez ^6^ ao reinterpretar Fanon na construção do conceito de “Améfrica Ladina”. Como mencionado anteriormente, também não se descarta o fato de que boa parte das estruturas sociais mais gerais que atravessam não só a Sociologia como também seus objetos na contemporaneidade são as mesmas (ou quase as mesmas) daquelas responsáveis pelas condições de produção dos chamados “clássicos” em questão (como - mas não só - os trabalhos de Marx, Durkheim e Weber, que no Brasil se constituíram como os três grandes fundadores do que se pôde chamar de Sociologia a partir do início do século XX). A Sociologia reinterpreta, assim, seus clássicos, produzindo o novo no interstício entre luz e sombra sobre objetos dinâmicos, como um corpo que nasce - ou que pare. É justamente esse tipo de movimento analítico que ela opera e que a permite capturar, descrever e procurar compreender fenômenos sociais notadamente marcados por dinâmicas semelhantes, como descreveram autores como Marx ^7^ a partir de Hegel ou, mais contemporaneamente, Alain Badiou ^8^ ^,^ ^9^ com a noção de “evento” ou “acontecimento”.

Notadamente, para o interesse deste texto, tomo como um desses casos aquele dos objetos sociológicos ligados à esfera da vida social que convencionou compreender como “saúde” - uma categoria sempre em disputa - como propunha Cecília Donnangelo, precursora de princípios que se tornariam pilares para a área da Saúde Coletiva no Brasil ^10^ ^,^ ^11^ ^,^ ^12^. Dessa forma, pensar saúde como problema sociológico contemporâneo exige que nos perguntemos de que maneira os fenômenos entendidos como pertencentes à área da “saúde” nessa disputa desafiaram a produção de conhecimento sociológico nesse período; ao mesmo tempo, essa questão implica também em refletir sobre as respostas que a Sociologia ofereceu a esses enigmas, por meio da elaboração de novos problemas.

## Contribuições da/para a sociologia da saúde na contemporaneidade

A divisão da Sociologia em subáreas especializadas é uma das marcas do processo de sua institucionalização e da disciplinarização das Ciências Sociais e Humanidades da qual fala Heilbron ^3^. A sociologia da saúde pode ser entendida, nesse sentido, como herdeira direta desse processo, que foi deflagrado nas condições particulares do que chamei aqui de “contemporaneidade”. Assim, se é verdade que a saúde é um objeto que escapa à própria sociologia da saúde e permite investigações diversas em uma gama maior de subáreas da sociologia, é verdade também que ela se constitui como objeto particular e distinto a partir dessa criação. Sabemos, como propõe Andrew Abbott em *Chaos of Disciplines* [Caos das Disciplinas] ^13^, que a afirmação de uma disciplina ou área de pesquisa não é um processo mágico e nem desinteressado, já que serve como ferramenta para a disputa concreta entre grupos de pesquisadores em seu espaço de trabalho (garantindo, por exemplo, melhor acesso a recursos de pesquisa que raramente são distribuídos de maneira igualitária). Da mesma forma, Vieira-da-Silva ^12^ descreve, em diálogo com a teoria bourdieusiana, as tensões que produziram o campo da Saúde Coletiva no Brasil. Segundo a autora, um dos principais fatores que opunha os diferentes grupos que estavam disputando a construção do entendimento nacional sobre a saúde no Brasil da década de 1970 era justamente o posicionamento em relação à inserção das ciências sociais em saúde como parte da formação profissional. Nesse sentido, disputava-se também uma compreensão acerca da própria categoria “saúde”: de um lado, um ponto de vista que privilegiava uma interpretação sobre o corpo físico, supostamente natural, destacado das condições sociais que o produziam; do outro, uma abordagem que via como indispensável a articulação entre processos fisiológicos e sociais ^12^.

Também cabe pontuar que nenhuma disputa bem-sucedida é feita em um vácuo. Quer dizer, a saúde foi e é objeto de diversas abordagens disciplinares e subdisciplinares das Ciências Sociais e Humanidades, tanto antes quanto depois da consolidação de uma área notadamente reconhecida e legitimada como “sociologia da saúde”. Da mesma forma, essa compreende uma gama de objetos de interesse que muitas vezes extrapolam a própria ideia de “saúde” (como os embates jurídicos presentes nesse espaço, as relações de trabalho, ou ainda as discussões sobre educação médica ^10^ ^,^ ^11^ ^,^ ^12^). Então, a “saúde” é, sociologicamente dizendo, um espaço de disputa - inclusive em torno de sua definição e da consolidação de seus objetos de análise ^12^.

Por outro lado, a sociologia da saúde apresenta uma particularidade interessante para a construção de novos problemas sociológicos: suas principais categorias críticas, hoje, são as que estruturaram as Ciências Sociais e Humanidades disciplinares desde seu início por serem, ao mesmo tempo, categorias dicotômicas, binárias e organizadoras da epistemologia fundamental do pensamento europeu dito “ocidental”. As dicotomias natureza/cultura e corpo/mente se correspondem nessa matriz simbólica, orientando as ações de sujeitos, instituições e grupos, e configurando os contornos da vida social, assim como suas possibilidades. Não é um dado desimportante, então, a percepção de que a desestabilização dessas duas dicotomias - natureza/cultura e corpo/mente - vêm acompanhando a construção de problemas sociológicos contemporâneos ligados à esfera da saúde. Processos semelhantes ocorrem em áreas interdisciplinares ligadas a outras ciências sociais, como a antropologia da saúde, espaço de produção de conhecimento em que a própria noção de “cultura” foi capaz de informar e transformar uma série de reflexões substantivas sobre o corpo e os ditos “sistemas culturais” e “sistemas sociais” da saúde ^14^. Essas dinâmicas mudaram a maneira como saúde, doença, corpo, medicina, curandeirismos e outros fenômenos são compreendidos nas Ciências Sociais e Humanidades.

No que diz respeito ao diálogo entre Saúde e Sociologia, destaco dois movimentos analíticos que parecem marcar a abordagem contemporânea da sociologia da saúde, nesse sentido. O primeiro deles consiste em aprofundar análises sobre as estruturas sociais que atravessam os fenômenos de interesse da área. Tributário, em parte, por uma maior proximidade dos movimentos sociais com as universidades a partir da década de 1960 (apontada e discutida também por Heilbron ^3^, entre outros), esse tipo de abordagem passou a considerar indispensável a observação de fatores como gênero, raça, classe, geração, deficiência física, territorialidade e outros marcadores sociais da diferença nas pesquisas sobre fenômenos ligados à saúde ^10^ ^,^ ^11^ ^,^ ^12^. Em paralelo, o segundo movimento analítico que atualmente marca esse espaço de produção de conhecimento parece ser justamente reflexivo de certa epistemologia da saúde; ou seja, uma aplicação do entendimento sociológico de que a forma de conhecer algo está sempre em disputa ^12^. As pesquisas que se voltam a esse tipo de debate refletem, por exemplo, sobre a distinção entre categorias nativas e categorias sociológicas para abordar certos fenômenos (como no caso da categoria gênero versus mulher, discutida mais adiante), repensam propostas epistemológicas distintas (como no caso da discussão sobre uma sociologia da medicina *versus* uma sociologia na medicina ou, ainda, uma sociologia médica) e questionam as categorias discursivas operadas no espaço pesquisado como parte de relações de poder (como a própria ideia de doença ou saúde, as categorias de deficiência e normalidade, entre outras).

Como já pontuado anteriormente, claro que tanto uma forma de trabalhar os objetos próprios da área quanto a outra dependem de fenômenos concretos que provoquem deslocamentos particulares em relação à forma sociológica de conhecê-los, assim como o parto da pequena Nelly. Embora essa não seja uma revisão bibliográfica sobre o tema, procuro pontuar a seguir alguns dos principais fenômenos que provocaram certa instabilidade para a sociologia da saúde na contemporaneidade, refletindo, na medida do possível, acerca dos ganhos sociológicos desses para a sociologia num geral.

Nas Ciências Sociais e Humanidades como um todo, desde a segunda metade do século XX, a oposição dicotômica entre natureza e cultura (e suas derivadas corpo/mente, razão/emoção etc.) vem sendo duramente questionada. A produção sociológica de intelectuais ligadas a movimentos políticos e sua crescente proximidade com a universidade possibilitou - como no caso da fundação dos estudos de gênero ^15^ - novas indagações sobre o conjunto de ferramentas analíticas até então disponíveis nas Ciências Sociais e Humanidades, em geral, e na Sociologia, em particular. O corpo, campo de batalha do movimento negro e do movimento feminista, se tornou, nesse sentido, não apenas objeto privilegiado de análise na teoria social de forma mais ampla, mas também passou a ser entendido como dispositivo/ferramenta para a compreensão de diferentes fenômenos sociais, notadamente aqueles ligados à saúde. No senso comum, a divisão natureza/cultura corresponde a uma dicotomia entre, de um lado, um domínio da natureza imutável e destinado geneticamente (corpo/emoção), e, do outro, um domínio da cultura moldável, adaptável e posterior aos limites da natureza (mente/razão). Os questionamentos colocados pelas Ciências Sociais e Humanidades e seus novos dispositivos teóricos contemporâneos criaram tensão em relação a essa suposta divisão. Exemplos marcantes para a contemporaneidade são o conceito de gênero, como discutido sobretudo por Rubin ^16^, Scott ^17^, Butler ^18^ e Haraway ^19^, ou a elaboração conceitual da raça como sistema simbólico de dominação da branquitude, como propõem Fanon ^4^ e Gonzalez ^6^, já citados, mas também Bento ^20^, Alves ^21^, Schucman ^22^ e outras. Esses são os dois exemplos (os conceitos de “gênero” e “branquitude”) que guiam as reflexões seguintes deste ensaio.

Uma marca significativa do período que convencionei neste ensaio se chamar “contemporaneidade sociológica” é a presença incisiva das disputas pela política sexual nas universidades e na sociedade em geral. As práticas sexuais e a sexualidade, assim como o que hoje chamamos de “gênero”, foram ideias catalisadoras de diversas transformações profundas em diferentes países a partir, especialmente, das décadas de 1960 e 1970. No Brasil, por exemplo, o fim do estatuto da mulher casada e a lei do divórcio foram consequências concretas desse tipo de disputa. A fundação do movimento de travestis em reação à Operação Tarântula ^23^ e outras formas de perseguição violenta da ditadura militar a esse segmento da população também são fenômenos que ilustram essa relevância ^24^. Na década de 1990, a epidemia de HIV e aids fez com que as políticas de saúde precisassem ser repensadas de maneira urgente, tratando a sexualidade e o gênero como assuntos indispensáveis para compreender os diversos fenômenos sociais novos deflagrados ali, indo do debate sobre casamento e herança entre casais homossexuais à biossegurança em práticas sexuais devido à transmissão de infecções sexualmente transmissíveis (IST) ^25^ ^,^ ^26^. A sociologia que pretendia analisar a saúde se viu, assim, confrontada com a maneira como marcadores sociais da diferença participam da produção do corpo, criticando os discursos biomédicos que pressupõem um “corpo puro”, “anterior” à dimensão simbólica ^18^. De maneira semelhante, de Virgínia L. Bicudo ^27^ à Lélia Gonzalez ^28^, a intelectualidade negra estabelecia as bases para se refletir sobre a maneira como a produção social do corpo e a produção social do sujeito estão imbricadas no tecido das relações sociais. Essas contribuições também foram base para questionamentos complexos sobre as possibilidades ferramentais das Ciências Sociais e Humanidades diante, de um lado, do desafio de conciliar a separação analítica das estruturas sociais e simbólicas que distribuem poder (como gênero, raça, classe, território etc.), e o fato indiscutível de que essas estruturas são experimentadas pelos sujeitos sociais de forma integral, ou seja, na interação e interseção.

Quer dizer, se num primeiro momento a “sociologia da saúde” era uma sociologia médica e buscava analisar principalmente os responsáveis pela produção discursiva sobre a saúde, tomando-a como um dado e não como uma categoria em disputa, foi na contemporaneidade sociológica que as tensões em relação a essa visão se afirmaram como de fato parte da análise sociológica sobre esse tipo de fenômeno. Enquanto a sociologia médica passou a ser entendida cada vez mais como parte da sociologia das profissões ou do trabalho (ou, ainda, da sociologia das elites, no caso do Brasil), hoje é possível dizer que a sociologia da saúde deve ser entendida como uma forma particular de sociologia da produção social do corpo. Os estudos de gênero e estudos *queer* notadamente contribuíram com essa transformação ao indagarem de que modo a própria categoria “saúde” é mobilizada como um dispositivo de poder. As experiências de pessoas intersexo ^29^ e transgêneras ^30^, sistematizadas em bibliografia mais recente (sobretudo a partir da década de 2000, no Brasil e fora dele), têm identificado categorias como “poder médico”, por exemplo, como uma possibilidade para compreender contradições, violências e vivências ocorridas no seio das relações sociais alinhavadas pela ideia de “saúde” ^31^. Não obstante, é justamente na maneira como as ideias de “saúde” e “natureza” se intersecionam, passando pelo “corpo”, que se configura um sistema de poder, no sentido atribuído por Foucault ^32^ ^,^ ^33^ a essa categoria. O poder, para o autor, é um exercício constante, pulverizado nas relações sociais e que afeta a produção simbólica coletiva, ao passo em que produz sujeitos que atuam em prol de sua manutenção.

Quando entramos naquele centro obstétrico, minha amiga Evelyn e eu, uma segunda pergunta era repetida, causando estranhamento com a resposta da gestante: “é menino ou menina?”. Quando ela dizia que não sabia, porque não lhe importava a genitália da criança, as paredes do hospital do Campo Limpo pareciam ruir. Thomas Laqueur ^34^ mostrou que “sexo biológico” é essencialmente uma categoria simbólica - e não biológica - examinando documentos históricos da medicina europeia. A genitália e as ideias de “sexo” associadas a ela foram tratadas de maneiras diferentes e sem linearidade alguma ao longo dos últimos milhares de anos pelos povos de matriz simbólica ocidental ^34^. Nada que antropólogas como Rubin também não tenham apontado, questionando por que seres humanos considerariam corpos com pênis e corpos com vagina como opostos se na natureza não haveria nada mais semelhante a um corpo humano do que outro corpo humano, independentemente de sua genitália ^16^. O conceito de gênero, que aparece primeiro como “sistema sexo/gênero” no artigo de Rubin ^16^, escrito e publicado por ela ainda como mestranda, desestabilizou a noção de que “sexo masculino” e “sexo feminino” seriam categorias descritivas de uma natureza supostamente inquestionável. 

Esse giro analítico das Ciências Sociais e Humanidades, exemplificado aqui pelos estudos de gênero (mas não limitado a eles), tem e teve impacto direto na maneira de interpretar diversos fenômenos ligados à saúde. Ora, se a ideia do senso-comum sobre a saúde parte de uma pressuposição de “natureza”, como já apontado, a desestabilização dessa ideia evidentemente disputa o entendimento do que seja “saúde”. Como discutido em obras relativamente recentes como *After Nature* ^35^ , manifesto ciborgue [*Simians, Cyborgs, and Women: The Reinvention of Nature*] ^36^ e *Manifesto das Espécies Companheiras: Cachorros, Pessoas e Alteridade Significativa* ^37^, a natureza (no caso dos estudos de gênero, mas também das Ciências Sociais e Humanidades em geral) passou a ser entendida também como um dispositivo discursivo de poder, que faz parecer imutável o produto da estrutura de dominação em prol da qual atua. A saúde, então, se consolida para a sociologia contemporânea como categoria socialmente disputada e construída, por meio da qual diferentes agentes operam as relações de poder e conflito que constituem e organizam o tecido social no contexto da modernidade capitalista. Nesse contexto, gênero e raça se consolidam como duas grandes contribuições teórico-analíticas na construção da saúde como problema sociológico contemporâneo.

No que tange ao gênero, as categorias de “sexo masculino” e “sexo feminino” são as primeiras a incidirem sobre o corpo, ainda na gestação ou, no máximo, imediatamente após o parto. A definição de pertencimento a cada uma dessas categorias é uma definição médica visual, ou seja, uma pessoa designada pela sociedade como competente para tal define, em critério visual, qual o lugar daquele recém-nascido na ordem sexual e de gênero da sociedade. Em sociedades de matriz simbólica ocidental como a nossa, essa é uma marca médica carregada no corpo por cada um de seus membros, tão antiga em sua história pessoal quanto a classificação racial. Assim como o gênero, a “raça” ou “cor da pele” é definida na maior parte das vezes pelas equipes de saúde diante do nascimento, a partir da observação do tom de pele da pessoa parturiente e, quando se sabe, da outra pessoa genitora. Trabalhos antropológicos como *O Corpo da Nação: Classificação Racial e Gestão Social da Reprodução em Hospitais da Rede Pública do Rio de Janeiro*, de Corossacz ^38^, mapearam essa prática e observaram que os critérios são subjetivos, partindo de pressupostos racistas antigos sobre o funcionamento da dita miscigenação racial num discurso supostamente genético.

Até o momento do parto, além disso, o corpo que gesta também já terá passado por uma série de experiências marcadas também pela classe - como mostram dados sobre violência obstétrica, acesso a pré-natal e serviços de saúde ^39^, entre outros temas caros à interseção entre a sociologia da saúde e os estudos de gênero, raça e classe. A própria definição social de que gestar e parir não pode ser um trabalho legítimo e remunerado, conforme discute Sophie Lewis em *Full Surrogacy Now: Feminism Against the Family* [Barriga de Aluguel Já: O Feminismo Contra a Família] ^40^, já delineia a produção social dos corpos e o conjunto de relações em que são produzidos. Em sua tese de doutorado, a cubana Yarlenis Mestre Malfrán ^41^ mostrou a maneira como a regulamentação do acesso à fertilização in vitro no sistema de saúde de Cuba reproduzia, ao menos antes do novo código de famílias do país, a exigência heterossexual, monogâmica e cisnormativa que embasa a noção de “família”, articuladora principal de gênero, raça e classe, conforme pontuo na seção seguinte do texto.

## Corpo, saúde, Estado: extrapolando o problema sociológico

Da mesma maneira que o sistema de gênero tem em categorias como “homem” e “mulher” dispositivos de poder, o racismo e o sistema de classes também operam com seu próprio conjunto de parafusos e dobradiças. Se no contexto do nascimento (evocado ao longo deste texto como ponto nevrálgico em que as relações sociais de saúde e suas contradições se explicitam) há uma distribuição desigual de poder que define o lugar de quem nasce na ordem social de gênero, isso também ocorre quando falamos de raça e classe. A “saúde”, espaço de definição muitas vezes violenta sobre as possibilidades dos corpos e dos sujeitos, ao ser operada a partir da noção de família encontra um pilar de sustentação para todos os rebites e para a fundação dos três sistemas simbólicos desiguais aqui discutidos: gênero, raça e classe). Raça e classe são categorias herdadas - ainda que não geneticamente. O gênero é uma pequena loteria, embora se herde, claro, algo dali também, seja no sentido social (como na ideia de performance de gênero de Butler ^18^) ou no sentido subjetivo. Nos três casos, são construções cotidianas da subjetividade, dependentes de um trabalho simbólico constante.

Embora esse trabalho simbólico apareça como relegado ao espaço doméstico e a divisão público/privado do senso-comum faça parecer que ele não é assunto do Estado, as evidências sugerem o contrário: esse é um trabalho fortemente regulado, desde a interdição de mais de um acompanhante/pai na sala de parto onde nasceu minha afilhada, até a legislação sobre casamento e herança entre pessoas registadas civilmente como tendo o mesmo “sexo biológico”. Indo além, trabalhos recentes, como *Couro Imperial: Raça, Gênero e Sexualidade no Embate Colonial*, de McClintock ^42^, e *Parenting Empires: Class, Whiteness, and the Moral Economy of Privilege in Latin America* [Impérios parentais: Classe, branquitude e a economia moral do privilégio na América Latina] ^43^, de Ramos-Zayas, mostram como noções raciais, notadamente a construção de parentalidade da branquitude, são prioridades para o Estado a medida em que produzem essencialmente a noção de “nação” da qual essa instituição depende.

De modo semelhante, pesquisas recentes mostram que a maneira como o Estado opera a categoria “família” é um indicativo importante de sua compreensão sobre esse trabalho concreto executado pelos sujeitos no espaço doméstico ^44^ ^,^ ^45^ ^,^ ^46^ ^,^ ^47^ ^,^ ^48^ ^,^ ^49^ ^,^ ^50^ ^,^ ^51^ ^,^ ^52^. Em um contínuo com as questões de saúde, toda a legislação sobre família é essencialmente uma legislação sobre o corpo, abordando que formas de atividade sexual e procriação são permitidas e quais os direitos de certos sujeitos sobre os corpos dos outros. Certos setores da sociologia da saúde e da sociologia da família estão, ainda, no instigante fenômeno contemporâneo da medicalização da parentalidade e da pedagogia doméstica - a ideia de que pais e mães precisam recorrer a especialistas (sempre de áreas médicas, biomédicas e próximas, como a psicologia) que deteriam o conjunto de saberes necessários a uma vida plena e feliz para a família e seus filhos ^50^. Esse tipo de discurso é enfatizado por organizações internacionais como a International Federation for Family Development (IFFD, Federação Internacional para o Desenvolvimento Família, Espanha), que atua oferecendo cursos e congressos de educação parental e familiar enquanto financia lobistas de “políticas pró-família” em diversos países do mundo, incluindo o Brasil. Não espanta, nesse sentido, que o governo de Jair Bolsonaro tenha não apenas criado um Ministério da Mulher, da Família e dos Direitos Humanos, mas também que um de seus carros-chefe tenha sido a transformação de um programa de educação parental em política pública - o programa Famílias Fortes, nascido no Ministério da Saúde de Michel Temer ^51^. Essa transferência da resolução de problemas sociais para a esfera privada ou moral da “família” vem sendo central para estudos recentes da sociologia da saúde que se debruçam sobre casos como o movimento antivacina, ou a política de saúde do governo Bolsonaro diante da pandemia de COVID-19. O posicionamento do Estado, ao operar esse tipo de transferência, aponta para uma reinterpretação em relação à sua própria função, mas também uma posição na disputa sobre o que devem ser políticas públicas ou Direitos Humanos, por exemplo ^44^ ^,^ ^48^ ^,^ ^49^. Por fim, mas não menos importante, cabe observar que esse tipo de transação se opera no meio político justamente apagando categorias analíticas como gênero, classe e raça do *modus operandi* em prol de categorias nativas do senso-comum que reforçam justamente a ideia de natureza, como “família” ou “mulher”, escondendo as estruturas que produzem os problemas que, ao menos em tese, o Estado deveria se dispor a enfrentar em prol de um bem coletivo ^52^.

É precisamente esse conjunto de normas que operava a experiência de minha amiga ao parir, e da pequena Nelly ao nascer. A determinação de gênero e raça, a experiência de classe no hospital municipal. A quantidade de acompanhantes e a pergunta insistente sobre a minha relação com a gestante. As experiências de diferentes casais de mulheres em que uma delas é registrada como “mãe adotiva” e a outra como “mãe biológica”. Os pais homossexuais que adotam seus bebês ou contam com gestação de substituição, e todo o pouco reconhecimento a essa atividade - além do estigma. Os abortos interditados, perseguidos e moralizados, colocando a gravidez no Brasil num lugar de obrigatoriedade inequívoca. Tudo isso nos recorda que a experiência humana de corpo é necessariamente atravessada pelas mesmas estruturas que socialmente o produzem.

Diante das paredes do hospital ruindo a cada pedido ou da informação pouco convencional da rede de pessoas que pariu Nelly, naquele outubro de 2022, estão os próximos desafios de uma sociologia - e de uma saúde pública - que está para nascer. Elaborar novos problemas sociológicos ou reelaborar a saúde enquanto problema sociológico será tão necessário como analisar o mundo, mas o tesão que faz pulsar o corpo e o cérebro de quem o questiona sempre estará, também, em transformá-lo.
